# Editorial: The promising role of non-invasive brain stimulation in neurocognitive disorders treatment

**DOI:** 10.3389/fnhum.2023.1340023

**Published:** 2023-11-30

**Authors:** Valerio Manippa, Marco Filardi, Anita D'Anselmo

**Affiliations:** ^1^Department of Education, Psychology, and Communication, University of Bari Aldo Moro, Bari, Italy; ^2^Department of Translational Biomedicine and Neurosciences (DiBraiN), University of Bari Aldo Moro, Bari, Italy; ^3^Center for Neurodegenerative Diseases and the Aging Brain, University of Bari Aldo Moro at Pia Fondazione “Card. G. Panico”, Tricase, Italy; ^4^Department of Psychological, Health, and Territorial Sciences, University “G. d'Annunzio” of Chieti-Pescara, Chieti, Italy

**Keywords:** neuromodulation, transcranial brain stimulation, neurophysiology, neurology, stroke, dementia, neurocognitive disorder

In this Research Topic, we focused our interest on the use of Non-Invasive Brain Stimulation (NIBS) techniques, such as transcranial magnetic and electric stimulation (TMS and tES), in neurocognitive disorders. These tools offer a safe means to manipulate brain activity without the need for surgery or anesthesia. NIBS tools are commonly used for studying brain functions, modifying cognitive and behavioral processes, and exploring applications in neuropsychiatric disorders in both human subjects and murine models.

Three of the papers included in our topic focused on the potential therapeutic effects of NIBS in post-stroke recovery. The systematic review of Gong et al. examined studies on the use of repetitive TMS (rTMS) for post-stroke cognitive impairment (PSCI). Twelve randomized controlled trials were included in the meta-analysis for a total of 497 PSCI patients. Both low-frequency (LF-) and high-frequency (HF-) rTMS applied to the dorsolateral prefrontal cortex (DLPFC) were effective in improving cognitive function and activities of daily living in patients with PSCI with any significant differences between LF- and HF-rTMS. The study concluded that rTMS represents an effective and non-invasive treatment for PSCI recovery, however, the authors suggest that more randomized studies with larger population samples and using multiple rTMS stimulation sites are needed to better understand the clinical efficacy of this treatment.

The general commentary of Li et al. highlights critical points of the systematic review of Wen et al. (2022) on the effect of rTMS in the treatment of post-stroke dysphagia. The points raised concern the missing of several randomized clinical trials, not included in the meta-analysis; the incorrect data extraction method that leads to large data heterogeneity; and the unclear selection of data indicators that increase measurement uncertainty and data error. In a subgroup analysis Li et al. found, in agreement with similar studies, that both LF- and HF-rTMS could significantly improve the swallowing function of patients with dysphagia. These results differ from those illustrated in the meta-analysis by Wen et al. (2022), who found that HF-rTMS is more effective than LF-rTMS in improving swallowing function in post-stroke patients. In conclusion, the authors suggest that readers should be cautious when considering the results of Wen et al. (2022) meta-analysis.

Finally, the methodological study of Carlson et al. investigated the strength of the electric field (EF) induced by 5 transcranial direct current stimulation (tDCS) montages targeting the motor system of children with cerebral palsy caused by perinatal stroke (PS). Simulations were completed on 32 typically developing controls, 21 patients with arterial ischemic stroke (AIS), and 30 with periventricular infarction (PVI). When anodal tDCS was applied to the lesioned cortex, EF strength values were higher for the AIS group compared to controls. Furthermore, montages with anodal stimulation on the lesional area were more sensitive to changes in the underlying anatomy (lesion and tissue volumes) than those with cathodal stimulation on the non-lesioned cortex. The results suggest that EF tDCS simulations, considering the peculiarities of the brain architecture after PS, are suitable for planning personalized tDCS rehabilitation interventions.

The other two papers focused on the effect of tES on neurological samples. The research of Han et al. investigated the impact of high-definition (HD)-tDCS on patients with Disorders of Consciousness (DOC). Nineteen patients received 10 anodal HD-tDCS sessions targeting the left DLPFC. Based on Coma Recovery Scale-Revision (CRS-R) changes, they were categorized as responsive (RE) or non-responsive (N-RE). In the RE group, HD-tDCS treatment led to a significant increase in resting-state electroencephalograph (rsEEG) power spectral density (PSD) in the 6–8 Hz frequency band in the posterior region. Single sessions increased PSD in the 10–13 and 13–30 Hz bands. A positive correlation was observed between changes in PSD values in the posterior region and CRS-R scores after single HD-tDCS sessions. The study concluded that repeated anodal HD-tDCS of the left DLPFC can enhance clinical outcomes in DOC patients, modulating cortical excitability by influencing the posterior region's PSD.

On the other hand, Cappon et al. explored the feasibility and effectiveness of a tele-supervised home-based transcranial alternating current stimulation (tACS) protocol in 8 Alzheimer's disease patients (ADs). The pilot study consisted of an acute phase, in which ADs underwent daily 20-minute sessions of home-based 40Hz-tACS over the left angular gyrus for 14 weeks, a 3-month hiatus with no stimulation, and a taper phase with 2–3 tACS sessions per week for 3 months. While high adherence was seen in all the intervention phases, side effects occurred in 33.4% of sessions, with severe effects occurring only in 1.2% of sessions. From a clinical standpoint, all participants showed memory improvement at the end of the acute phase (assessed with the Memory Index Score) and this improvement persisted during the hiatus phase. In contrast, the intervention showed no effect on global cognitive functioning, as assessed by the Montreal Cognitive Assessment. Three participants who underwent rsEEG before and after the acute phase reported a decreased theta/gamma ratio (an AD neurophysiological marker) in the angular gyrus. This study suggests promising results for home-based neuromodulatory interventions in terms of safety and feasibility. If confirmed in larger studies, these findings could significantly impact the clinical and therapeutic management of ADs.

Finally, two papers explored a challenging tACS application and the molecular rTMS mechanism of action, respectively. With his mini-review, Takeuchi suggests that personalized tACS combined with a physical program can restore disrupted brain oscillations reducing patient-reported pain. Chronic pain is linked to altered neural oscillations (i.e., increased theta, and decreased alpha, and beta oscillations) that can be modulated through tACS. Further, tACS could be used to induce inter-brain synchrony to enhance therapeutic alliance. Factors like pain empathy, interpersonal touch, and clinician-patient relationships are reflected in inter-brain communication inducing analgesic effects. The application of hyper-tACS (i.e., tACS applied simultaneously to two individuals) over M1 of the clinician and the patients, might facilitate clinical compliance in pain therapy and pain control, but the application of such protocol in clinical settings remains a challenge due to ethical considerations. In conclusion, tACS for chronic pain control is a promising but evolving field, necessitating further refinement and deeper insights.

Weiler et al., instead, explored the molecular basis of the rTMS mechanism of action using three experimental rat models (*in vitro, ex vivo*, and *in vivo*) and genome-wide microarray analysis. In the *in vitro* experiment, embryonic hippocampal neurons from E18 Sprague-Dawley rats received either 1 Hz, intermittent Theta Burst stimulation (iTBS), or sham stimulation. In the *ex vivo* experiment, hippocampal slices from 12 Long-Evans rats (4 young, 4 aged unimpaired, and 4 aged impaired) underwent either 1 Hz, iTBS, or sham stimulation, with RNA collected after 2 h of stimulation. Finally, in the *in vivo* experiment, 8 young and 8 aged rats received the same stimulation protocol and were sacrificed 48 h post-stimulation, with areas of the neocortex and dorsal hippocampus frozen and stored. In rat hippocampal neuronal cultures, rTMS induced widespread changes, upregulating or downregulating the transcription of genes involved in complex neural processes, including multiple GABA and glutamate receptors, as well as genes associated with learning and memory-related plasticity. Moreover, both the *ex vivo* and *in vivo* experiments showed that rTMS produced a substantial reduction in transcripts involved in the immune and inflammatory processes. This study represents one of the first investigations into transcriptional changes induced by rTMS, providing a foundational characterization for subsequent experimental investigations.

## Author contributions

VM: Writing—original draft, Writing—review & editing. MF: Writing—original draft, Writing—review & editing. AD'A: Writing—original draft, Writing—review & editing.
